# Interviewing Male Survivors of Sexual Violence and Abuse: Ethical and Methodological Considerations

**DOI:** 10.1177/08862605221093683

**Published:** 2022-05-07

**Authors:** Siobhan Weare, Joanne Hulley

**Affiliations:** 1 The Law School, 4396 Lancaster University, Lancaster, Lancashire, UK; 2 School of Human and Health Sciences, 4013 University of Huddersfield, Huddersfield, West Yorkshire, UK

**Keywords:** male survivors, sexual violence and abuse, interviews, ethics, methods, gender

## Abstract

Existing research explores ethical and methodological considerations associated with interviewing men, including male survivors of domestic abuse, and interviewing female survivors of domestic and sexual abuse. However, there is no comparable body of research that specifically considers interviewing male survivors of sexual violence and abuse. Reflecting upon our experiences of interviewing 32 male survivors of female-perpetrated sexual violence, we critically discuss four key ethical and methodological issues that arose; the challenges around recruiting male survivor participants; the interview process; the impacts of gender dynamics between interviewers and participants; and the importance of research to participants. Based on our reflections we make a number of recommendations for scholars who are conducting future research with male survivors of sexual violence and abuse.

## Introduction

It is estimated that 155,000 men aged 16–74 years experienced sexual assault (including attempts) in England and Wales in the year ending March 2020; a prevalence rate of approximately 1 in 100 men (Office for National Statistics, 2021). Despite the prevalence of male sexual victimisation, relatively few research studies have interviewed male survivors about their experiences (exceptions include e.g. Ralston, 2020). It is therefore perhaps unsurprising that no scholarship, to date, has explored the specific ethical and methodological issues that arise, and should be considered, when interviewing this group of men. In this article, we reflect on interviews that we conducted with male survivors about their experiences of being forced-to-penetrate a woman, and the key ethical and methodological issues that arose. More specifically we discuss; the challenges around recruiting male survivor participants; the interview process; the impacts of gender dynamics between interviewers and participants; and the importance of research to participants.

## Existing Literature

No existing methodological literature looks *specifically* at the issue of interviewing male survivors of *sexual violence*. The broader context of interviewing men (as a homogenous group) has received some, albeit limited, attention, with a primary focus on displays of masculinity by male participants and the impacts this can have, both on the interview process, and the data collected (Pini, 2005). Consideration has also been given to the gender dynamics that may arise in the interview process, particularly where interviewers are women, and concerns around power imbalances, sexism, and harassment towards female researchers have been noted (e.g. Lee, 1997; Schwalbe & Wolkomir, 2001; Pini, 2005; and Blagden & Pemberton, 2010). Within this context, Robb (2004, p. 404) highlights the importance of being ‘reflexively aware of the influence of … [gender] dynamics on the co-construction of narratives both in the interview itself and in its interpretation after the event’. The importance of reflexivity when conducting research with men more broadly has also been highlighted. For example, being reflexive ‘about behaviour or presence that might limit men’s sense of personal confidence and willingness to discuss their fears, anxieties, and experiences’ (Hutchinson et al., 2002, p. 48).

In relation to men as interview participants, the experiences of interviewing *specific groups of men*, for example fathers (Gatrell, 2006) and male sex offenders (Blagden & Pemberton, 2010), have been reflected upon, as well as the experiences of interviewing men about *specific subjects*, for example, fatherhood (e.g. Robb, 2004) and health issues (e.g. Brown, 2001). Within these contexts observations have been made about the difficulties associated both with recruiting male participants, as well as eliciting discussions with men around certain topics (Brown 2001, p. 190). Most recently, Douglas et al. (2021) have reflected upon conducting focus groups with male victims of partner abuse, and in particular the use of technology within this context. They highlight male victims of partner abuse as a ‘hard to reach’ population, something which is exacerbated by a lack of existing structures to support male victims. They conclude that technology maximises engagement with male victims within the context of focus groups. Whilst some of the discussions within this body of research may be *relevant* to scholars working with male survivors of sexual violence, none of the existing literature *directly addresses interviewing men* about their experiences of sexual abuse and violence.

There is also a body of scholarship that focuses on interviewing women survivors of sexual and domestic abuse, which is undoubtedly useful to draw upon when interviewing male survivors. This body of feminist scholarship highlights the importance of engaging with women as gendered beings within every stage of a research project. As Fontes (2004, pp. 160–161) notes; ‘ignoring gender and all of its implications in research design and analysis is unjust because it fails to note the importance of gender-based power imbalances’. The context within which women are participating in such research projects must also be acknowledged; in particular a recognition of the fact that ‘women who agree to speak about their victimisation are speaking out in a societal context of disbelief, fear, and shame’ (Fontes, 2004, p. 143).

A comprehensive account of major challenges that may emerge for researchers and female survivor participants when exploring violence against women and girls (VAWG) is provided by Fontes (2004). She cautions against replicating violations of trust in the research process, given that the topic under research includes both a physical boundary violation and potentially the violation of an intimate relationship. Fontes (2004) also highlights the importance of ensuring women’s consent to participation, recognising that issues of power, including for example the researcher’s authority, may (inadvertently) coerce respondents into participation.

Existing literature also emphasises the crucial nature of ensuring that strategies are in place during the interview process to protect women from further victimisation. For example, by forewarning respondents that steps will be taken to either terminate the interview, or switch to another topic of discussion if the interview were to be interrupted (Ellsberg & Heise, 2005, pp. 39–40). Other suggestions for minimising risks for female survivor participants include developing a variety of interview sites, ensuring participant anonymity at all stages of the research process including dissemination, and considering the potential for re-traumatisation of participants (Fontes, 2004, pp. 154–165). To minimise this potential for re-traumatisation, Ellsberg and Heise (2005) note the importance of signposting female survivors of violence to support services (p. 40), as well as the necessity of interviewer training on ‘how to identify and respond appropriately to symptoms of distress’ (p. 40). They also highlight the benefits associated with involving organisations ‘that carry out advocacy and direct support for survivors of violence’ (p. 45) within research projects, which include guiding the study design, advising on question wording, and publicising and applying the research findings (p. 45).

These areas of scholarship are undoubtedly useful when considering methodological and ethical issues around interviewing male survivors of sexual violence. Indeed, it will be seen throughout this article that we draw upon much of this literature. However, as a topic in its own right, an in-depth exploration of ethical and methodological considerations relevant to interviewing male survivors of *sexual violence* is missing from the literature. Therefore, by reflecting upon our experiences of interviewing male survivors of female-perpetrated sexual violence, this article makes an original contribution to knowledge and goes some way to filling a significant gap in both scholarship and understanding in a previously undiscussed area.

## The Study

The research study underpinning the discussions in this article was conducted between May 2018 and July 2019 and explored the experiences of men who had been forced-to-penetrate women. The term ‘forced-to-penetrate’ (FTP) is used in cases where a man is forced-to-penetrate a woman’s vagina, anus, or mouth with his penis, and without his consent (Weare, 2018, p. 110). These cases are not recognised as rape under UK laws, and thus cannot be labelled as such (see, Sexual Offences Act 2003, section 1; Sexual Offences (Scotland) Act 2009, section 1; and Sexual Offences (Northern Ireland) Order 2008, Article 5). The study focused on the contexts within which men’s FTP experiences took place, the impacts it had on them and their lives, and their engagement with support services and the criminal justice system.

Survivors Manchester (n.d.) were our partners in the research study (reflecting similar approaches highlighted within feminist methodological scholarship, e.g. Ellsberg & Heise, 2005). They are a survivor-led/survivor-run voluntary organisation based in Manchester UK, that specifically supports male survivors of sexual abuse and rape through one-to-one counselling sessions and group support. They also provide an Independent Sexual Violence Advisor service. Our partnership with them was deeply embedded within the project from its inception, with them providing advice around the conducting of interviews, and being involved in the publication of outputs. As a support organisation working specifically with male survivors of sexual violence, partnering with Survivors Manchester supported us in centralising the gender-based experiences and needs of our male survivor participants throughout the project (Fontes, 2004).

Prior to commencing the study, ethical approval was gained from the authors' Research Ethics Committee. 32 interviews were conducted, either face-to-face at Survivors Manchester’s premises, via telephone, or via Skype, with men who self-identified as having been FTP a woman. When advertising the study it was explained that FTP cases encompass *any* and *all* cases where a man engages in penile penetration of a woman without the man’s consent. Participants reported their FTP experiences occurred in a number of contexts including within an intimate abusive relationship alongside other forms of abuse, as a result of having their drink(s) spiked, and as part of being blackmailed about their sexuality (an overview of findings from the project can be found at, Weare & Hulley, 2019). The average age of participants, based on the 28 men who provided this information, was 42.9 years, with ages ranging from 24 to 66. In terms of sexuality, based on the 30 men who provided this information, most men identified as heterosexual (25), with two identifying as homosexual, two as bisexual, and one as queer. Minimal (and optional) demographic information was gathered from participants as the sample was purposive, rather than representative. In terms of geographic locations, participants came from England (26), Wales (3) and Scotland (1).

The length of the interviews varied considerably (between 30 and 180 minutes). Interviews were recorded and transcribed verbatim. The data then underwent thematic analysis (Braun & Clarke, 2006) with the researchers familiarising themselves with the data by each reviewing it independently by hand, before generating and comparing initial codes and themes. These themes and the project’s research questions were then reviewed and used to inform computer aided analysis and coding using the NVivo software.

## Ethical and Methodological Considerations

Whilst the original aim of the study was not to consider methodological and ethical considerations relevant to interviewing male survivors of sexual violence, these emerged during reflections and conversations on our experiences as researchers and those of our participants across the duration of the project. What follows therefore is a critical discussion of four key considerations and challenges that emerged when conducting this study. In our discussions we include our own critical reflections, as well as making reference to relevant literature from cognate and related areas of study.

### Recruiting Participants

Existing research on interviewing as a research method has highlighted gender variation in the recruitment of participants. For example, it has been noted that it can be difficult to recruit male participants in relation to certain research topics, for example, health-related research (Brown, 2001, p. 190). As such, we anticipated that male survivors would be a hard-to-reach group (Douglas et al., 2021), not least because they were being asked to share their experiences of a hidden form of sexual violence (FTP cases) that had not been previously researched in this way (i.e. via interviews) within the UK. Moreover, we were aware that stereotypical constructions of masculinity and male sex roles that, for example, depict men as ‘strong, powerful, and unable to be sexually dominated’ (Ralston, 2020, p. 129) may serve as barriers to disclosure about their experiences.

However, the anticipated challenge in recruiting male survivors was not realised, with around 70 men expressing an interest in participation, and 32 men ultimately being interviewed. This was more than the 25 interviews that were originally planned and promised to the project funder. We believe that our success at ‘over-recruiting’ male survivors can be attributed to a number of factors. A project website was created which provided detailed information about the study and what involvement would entail, signposted potential participants to support services, and provided basic information about the research team. The principal investigator’s (PI) email address was provided so that interested participants could get in touch, and any questions could be answered. Social media, particularly Twitter, played a large role in recruitment, with links provided to the project website. This was particularly effective in raising the profile of the project both nationally and internationally, with hundreds of likes, shares, tweets and re-tweets. Traditional media, that is, newspapers, magazines etc., were also used to publicise the project and encourage participation. For example, Weare was interviewed by ‘You Magazine' (a supplement in the Mail on Sunday) about the project (Moore, 2018). Some of the men directly referenced reading or hearing about the project as a result of media coverage.

Our partnership with Survivors Manchester meant that they were able to circulate information on the study within their networks. Other third sector support services (voluntary and community organisations and charities) and professionals working in the area also distributed details of the project as they felt appropriate. Survivors Manchester also had a role in providing ‘triage’ support for participants (discussed in further detail below). This, alongside clear signposting to a variety of support services prior to, during, and following participation, reassured survivors about the ethical nature of the project.

Weare had also conducted previous research in this area in 2016–17 which had involved 154 males completing an online survey about their most recent FTP experiences (Weare, 2017). Several of the men who had participated in this study indicated their willingness to participate in future research and provided their contact details accordingly. The findings from this first project received a significant amount of media attention and resulted in other male survivors getting in touch to be involved in future research. The success of this first study perhaps enhanced the sense of Weare being somebody who was ‘trustworthy’, and whom male survivors would be comfortable discussing their experiences with.

The fact that this study was the first of its kind in the UK, and thus represented the first opportunity for men to share their FTP experiences in a research interview context may also have been a relevant factor. Previous research has indicted that participating in research on sexual violence can have numerous benefits for survivors, which ‘seem to outweigh the immediate distress that accompanies discussion of painful experiences’ (Draucker, 1999, p. 161). These benefits include catharsis, empowerment, self-acknowledgement (validation of self-worth), self-awareness, sense of purpose (helping others), healing, and being heard (Hutchinson et al., 1994). Many of the men who participated in this study conveyed their feelings of gratitude and empowerment (discussed in more detail below), and thus it is possible that personal empowerment was another motivation for men’s participation in this research.

Whilst, as noted above, approximately 70 men indicated an initial interest in being involved, just under half of these men (32) were actually interviewed. Some men chose not to engage with us further after our initial contact. We only followed up with potential participants once to avoid risk of harm to survivors, and if we received no response, we assumed they were no longer interested in being involved. Several men arranged interview dates but withdrew prior to the interview taking place because they felt it would be too difficult to discuss their FTP experiences, or that it would have a detrimental impact on their mental health. It also transpired that several men were located internationally and were therefore not eligible for participation, with the study being limited to participants based in the UK. The majority of these men indicated they would like their contact details to be kept by the research team, should the opportunity for a future international research study occur.

One challenge that arose in participant recruitment related to a lack of access to technology for potential participants. Interested survivors were asked to contact the research team via email. However, a small minority of those interested did not have regular access to the internet, and needed to visit their local library or internet café to send/receive emails. To enable participation by these men, we asked them to provide us with their addresses, which allowed us to post out hard copies of the information sheet and consent form, with a pre-paid envelope for its signed return. Address information was not kept on file. We also asked for their telephone numbers so that we could contact them to arrange an interview time, and also conduct the interview by phone. Whilst this was not something that we had planned for, our approach worked well and enabled these men to successfully participate in the study.

### The Interview Process

Prior to their interviews, participants were provided with a detailed information sheet which outlined the project’s aims, and explained the interview process. Ethical issues of anonymity and confidentiality were addressed, informing respondents that audio files would be transferred to researchers’ password protected computers in encrypted files. It was also explained that recordings would be deleted following transcription to further protect participants’ identities and interview transcripts would be anonymised, with all identifying information removed. Survivors who participated via phone or Skype were also told about a safeguarding procedure we put in place to mitigate potential risks in relation to their participation, whereby if the connection was disconnected we would work under the assumption that this was initiated by the participant and would not call them back. We explained that we would then wait for 15 minutes in case they wished to call us back, or alternatively they could email us to call them again at a different time. This recognised that participants may still be in abusive relationships and their call may have been interrupted by their partner, or that they no longer felt able to continue the interview for any other reason (a similar approach is recommended by Ellsberg & Heise, 2005, pp. 39–40).

Given the sensitive nature of the research, participants were told prior to the interview that they did not need to answer any questions with which they were uncomfortable. It was repeatedly made clear that they could take a break if necessary, they could end the interview at any time, and they had the right to withdraw from the research up to two weeks after the interview without providing a reason for doing so. In light of the interview focus and the potential of evoking distressing memories and painful emotions, the men were informed how unsafe it might be to disclose without immediate support. Prior to and following the interview they were signposted to support services, with details also hosted on the project website for long term access. These included face-to-face services (e.g. the directory on the Male Survivors Partnership website), online and telephone support (e.g. the National Male Survivor Helpline and the Samaritans) and self-help resources (via the NHS website). The variety of sources signposted to participants ensured that they were able to access some form of support 24 hours a day.

It should be noted here that we felt it was particularly important to signpost participants to a variety of support because specialist support services for male survivors may be less visible or less accessible, for example, due to geographic location. This was a sentiment shared by several participants who were not aware that specialist support services for male survivors existed. For example, Participant 30 explained; ‘I mean I wasn’t aware that these people existed until I was in contact with you.’ Survivors Manchester also agreed to provide a ‘triage’ service, whereby they provided same-day specialist support to participants who requested or required it following their interviews. This was available either face-to-face (if interviews were conducted on their premises), or remotely via telephone. This ensured that any men who needed immediate specialist support were able to receive it, safeguarding them and helping to minimise potential re-traumatisation from their participation in the research.

A flexible approach to the interviews was employed**.** Men were given the option of participating via several formats – face-to-face at Survivors Manchester, via telephone, or via Skype. This helped to ‘minimise the risk of exposure and assure safety’ (Fontes, 2004, p. 154), by allowing participants to choose the most appropriate interview format based on their personal circumstances. The majority of interviews were conducted via telephone. This format was perhaps favoured for the relative anonymity provided by the lack of face-to-face contact. Participants sometimes explained during their interviews why they had chosen the interview format they had. For example, Participant 30 who was interviewed face-to-face at Survivors Manchester explained:

I had to do it face-to-face. I forget things on the phone you see, I’d have forgotten to say this, I’d have forgotten to say that, you know. I mean I’ve got a stammer anyway but it would be much worse if I’m not face-to-face.

The interviews were semi-structured, using ‘an open framework that allows focused yet conversational communication [and allows] both the interviewer and the person being interviewed some flexibility to probe for details or to discuss issues that were not included in the interview guide’ (Ellsberg & Heise, 2005, p. 130). The interview schedule was intentionally kept broad so that the diversity of men’s experiences could be captured through their own words.

We wanted to create a space in which participants felt they could share their experiences as *male survivors* in as much detail as was possible for them, including expressing ‘themselves openly about emotional issues’, something which is stereotypically associated ‘with threatening the boundaries of the masculine self’ (Robb, 2004, p. 403). Therefore, we sought advice from Survivors Manchester on the wording of interview questions to maximise participant engagement, whilst minimising potential harm, as well as the various interview approaches that could be taken. We took a reflective and reflexive approach to the interview process, which resulted in our approach changing over the course of the project. We began the first few interviews by asking basic demographic questions, for example, about participants’ age, the area of the UK they lived in etc., in an attempt build a rapport prior to discussing their FTP experiences. However, we found that contrary to the experiences of some other researchers (e.g. Ralston, 2020), participants preferred to get straight into sharing their stories (Draucker, 1999). As the interviews had been arranged days, or even weeks in advance, it is possible that participants had been considering how they would tell their stories and so were keen to share once the interview date arrived. We therefore altered our approach, and after checking that they were still happy for the interview to be audio-recorded, signposting to support services and asking if they had any further questions, we simply began by asking them to tell us about their FTP experience(s). We found this to be the most effective approach to take, with it almost being like ‘turning a tap on’ for many of the participants by asking this question; as if they felt they had been ‘given permission’ to share their story.

We found that once the men began talking, it was best to allow them to complete their stories uninterrupted (Bergen, 1993, p. 208), making notes on any points we needed to pick up on and saving these for discussion later in the interview. If there was a period of silence, we then asked specific questions about the men’s experiences, or asked questions clarifying some of the details they had already provided, as a means to continue the conversation (Bergen, 1993, p. 208). As a result of our change in approach, the demographic questions were moved and asked at the end of the interview. We concluded by asking participants how they were feeling, and reminded them of the support services available. The men were all asked whether they wished to receive a copy of the end-of-project report when it was published, which the vast majority did.

Existing research has noted that interviews with men can be brief and that men may be less expressive about their feelings and emotions (Brown, 2001, p. 189). However, we found that most of the men in our study were able and willing to discuss their experiences in detail, as well as express often complex feelings and emotions (albeit to varying extents). However, others struggled and required additional prompts or reassurance. Where this occurred one helpful tactic, as noted by Hutchinson et al., was to use ‘the method of countersuggestion’ and corroboration, where we sometimes drew upon ‘general data from previous interviews’ (Hutchinson et al., 2002, p. 50) to prompt participants to provide more detail. For example, ‘some of the other men we have spoken to have said X, did this happen to you?’, or ‘other men we have spoken to have also had similar experiences’. It was also the case that when some participants were sharing their stories they looked to have them corroborated or supported, and would ask us whether other men had had similar experiences. As noted by Elmir et al. (2011), topics which have previously remained silent may be experienced as ‘isolating’, and therefore this desire for experience validation was perhaps unsurprising, as many of the participants had either never discussed their experiences before, or had only told a few people. Moreover, several participants thought they ‘were the only man’ this had happened to, and so by asking about the experiences of other men, they were reassured and validated about their own. Following such reassurance, participants often became more open about discussing their experiences, allowing the interview to continue and develop further.

### Gender Dynamics between Interviewers and Participants

A body of scholarship has considered ‘how gender is performed by the interviewer/interviewee and considered the implications of this’ (Pini & Pease, 2013, p. 7) for research. In relation to interviewing men, the majority of scholarship has explored the gendered dimensions which exist when the interviewer is a woman, rather than a man (see e.g. Lee, 1997; Schwalbe & Wolkomir, 2001; and Pini & Pease, 2013). It is this scholarship that is most relevant to this paper as in this study both researchers were women. Much of the commentary has documented the negative experiences of, and challenges faced by, female researchers when interviewing men. For example, ‘inappropriate sexualising ... [as a way to] try to reassert control [which] can take the forms of flirting, sexual innuendo, touching, and remarks on appearance’ (Schwalbe & Wolkomir, 2001, p. 208), as well as other control strategies ‘such as seeking to direct the interview and interrupting’ (Pini & Pease, 2013, p. 7). Scholars have also reported having to listen to sexist, misogynist, and derogatory views by male interviewees which has silenced their own voices (Pini & Pease, 2013, p. 7) and left them conflicted about how their silence may provide ‘tacit support for’ such views (Pini & Pease, 2013, p. 10).

As female researchers we experienced inappropriate sexualising and misogyny in two of the 32 interviews that we conducted. Both of these interviews were ultimately excluded from the final dataset because they failed to meet the participation criteria of disclosing and discussing a FTP experience. In terms of participant behaviour, in one of these interviews the participant presented numerous hypothetical scenarios which included unnecessarily graphic and detailed sexual descriptions. In the other interview, the participant made a number of misogynistic comments and focused upon a false allegation he claimed was made against him by a woman. Our experiences during these two interviews can be contrasted with those in the other 30 interviews where all participants disclosed and discussed their FTP experiences in detail without such incidences. Upon reflection, we found it interesting that the two interviews which we excluded for not meeting participation criteria (i.e. disclosing and discussing a FTP experience), were the two within which we encountered some of the problematic behaviours experienced by other female interviewers. This perhaps indicates that these two participants had ulterior motives for wanting to be interviewed for the study.

Whilst many of the 30 study participants were (understandably) angry about their FTP experiences, the societal and cultural silence around their victimisation as men in the context of a focus on supporting female victims, and with the female perpetrators themselves (Weare & Hulley, 2019), none of them were overtly misogynistic or derogatory about women as a group. In contrast, when asked about how they would label their FTP experiences, several of them were concerned that they did not want to minimise, or be perceived as minimising, the experiences of other victims of sexual violence, particularly women. For example, Participant 20 explained; ‘You kind of feel like you’re reducing their victimhood, it’s like they’ve really suffered from this … it feels like it would cheapen their experiences and their emotions sometimes’.

In relation to other concerns raised within the literature around power and control strategies by male interviewees, this was not something that emerged as problematic within our 30 interviews. Power dynamics were fluid and varied between interviews, with us as researchers ‘being dominant at particular times and respondents being dominant at others’ (Reed, 2000, para.6.6). Indeed, whilst from the outside we may have looked like ‘knowledgeable researchers’ and have taken this role at various times, we recognised that in sharing their stories the participants were ‘knowledgeable informants’ (Reed, 2000, para.6.7) who were co-producers of the research. Therefore, it was important for us that participants asserted their own knowledge and power when sharing their experiences as male survivors. We privileged the meanings that men ascribed to their experiences (Anderson & Kirkpatrick, 2016), and allowed them to ‘take the lead’ in discussions. In some of the interviews participants asked for our perspectives on what they had discussed or issues they had raised. We did not feel this was done in a derogatory or negative way, nor to undermine us as female researchers. Any interruptions by participants were what we felt would be expected within conversations on a complex and emotional issue.

It is possible that being an all-women research team supported our access to participants and encouraged more detailed disclosures during interviews. This supports Lee’s (1997, p. 554) observation that ‘different gender identification might be … important in certain research projects.’ Indeed, it has been observed that men are more likely to be open about emotional issues with a female researcher (Robb, 2004, p. 404), and to confide in women about their experiences (Flood, 2013, p. 67), when compared to male researchers. This may be because when interviewing men, ‘women are researching in relation to another object, ‘men’; [whereas] men are doing so in relation to a similar object a category of which they are part’ (Hearn, 2013, p. 27). Male participants may therefore find it difficult ‘to express themselves openly … especially to other men [because it] risk[s] threatening the boundaries of the masculine self’ (Robb, 2004, p.403). Indeed, when discussing a topic such as FTP cases, where ‘masculine identities [may be] seen as being challenged … there is a sense of anxiety about losing power associated with those identities’ (Pini, 2005, p.212), which is more pronounced when ‘both parties to the encounter are male’ (Robb, 2004, p.402–3).

Whilst women may also be viewed as a ‘dangerous audience’ (Schwalbe & Wolkomir, 2001, p. 204) for men discussing topics relevant to their masculinity, there is also an ‘ease of rapport [and] empathy’ (Chandler, 1990, p. 127) stereotypically associated with women which may mean that they are perceived as a ‘safer’ audience to interact with in relation to emotionally sensitive information. These sentiments were reflected in comments made by some participants who noted that it was easier for them to discuss their FTP experiences with women than men. For example:

Now me personally, I’d rather speak to a woman. If it had been a man doing this I would have still spoken to them, but personally I’d prefer it to be a woman. That’s not the same for everybody – Participant 30.

[Discussed in the context of reporting to the police] I think possibly a first port of call for men who have experienced this would be to actually have a female officer listen to them … They’re better listeners. – Participant 19.

That is not to say however, that all participants felt this way, and indeed some explained that they had had very positive responses when discussing their FTP experiences with other men. For example, referring to his male friends, Participant 28 explained: ‘Three of the guys were absolutely fabulous and wasn’t what I was expecting at all, just absolutely brilliant’.

We are unable to comment on whether any potential participants were deterred from participating after finding out that both researchers were female. However, it is possible that some men may have wanted to avoid contact with female researchers given that the perpetrator of their FTP experience was female. Similarly, it is difficult to comment on whether the interview process and outcomes would have been different had we been a mixed gender or all-male research team. However, the potential need for the inclusion of a man on the research team was noted by one participant:

Maybe if you’ve got er, sounds really weird, a male member of staff that you might be able to get to do some of the interviews as well, ‘cause I had a thought while I was giving the interview what happens if it was still a raw thing and a bit hard for me, would I be able to talk to a woman about it? - Participant 9.

As an all-female research team on a project focussing on men’s experiences of sexual violence perpetrated by women, we were acutely aware of the potential impacts this could have on participants involved. Indeed, both before and during the interviews we considered the gender dynamics and influences that an all-female research team could have on the interview process and participants’ emergent narratives. Following the completion of the interviews, we also considered potential impacts of female researcher–male participant gender dynamics in relation to our analysis and interpretation of the data collected (Robb, 2004, p. 404).

Any researchers interviewing male survivors of sexual violence must be aware of the potential impacts of interviewer gender when designing the project, as well as conducting the research. Whilst not arguing that ‘male researchers should not interview men about issues that touch on masculine identity’ (Robb, 2004, p. 404), which sexual victimisation does, we would recommend that at least one of the interviewers is a woman. This reflects the sentiments of other academics and the experiences of our participants as noted above, as well as our own experiences. Regardless of the gender of the interviewer, challenges, albeit different ones, are likely to emerge and it is important that researchers are ‘reflexively aware of the influence of these kinds of dynamics’ (Robb, 2004, p. 404) on the research project as whole. Whilst female interviewers in particular should be aware of the specific issues noted by their peers (discussed above), based on our experiences we would suggest that they should not assume that they will automatically present themselves in the context of interviewing men about their experiences of sexual violence victimisation.

### Importance of the Research to Participants

The interviews sought to give a voice to male FTP survivors, to empower them, and to involve them as ‘co-producers of knowledge’ (Westmarland & Bows, 2019, p. 17). As researchers, we were careful to make the interview experience one that was non-judgemental, neutral, and conducted ‘in a safe and respectful environment’ (Elmir et al., 2011, p. 16). Reflecting the experiences of researchers who have interviewed female survivors, we found that our male participants ‘valued the opportunity to ‘tell their story’ and/or to gain greater understanding of their experience through the lens of the interview questions’ (Westmarland & Bows, 2019, p. 24). Indeed, the overwhelming feelings conveyed to us by participants were those of gratitude and empowerment. This is perhaps unsurprising when considered in the context of the historical silencing of all victims of sexual violence, and in particular the lack of previous research and discussion around FTP cases.

The benefits of research participation are widely noted in the literature. These include the empowerment of participants (Opie, 1992) through for example, opportunities for reflection on their experiences which may be cathartic and useful in obtaining closure, and opportunities to transform ‘their pain into helpful experiences for others’ (Fontes, 2004, p. 164). Therapeutic effects and feelings of relief and unburdening (Elmir et al., 2011) have also been noted. Several participants in our study indicated experiencing these, and other, benefits. For example, Participant 24 informed us that he had been:

looking forward to [participating] … it’s kind of something that’s put under the carpet and you never hear of it … but for somebody like yourself and your team to wanna get more information … it kinda feels like … what happened to me wasn’t in vain … I can then put it to rest … just sharing this with you today feels like it’s taken it off me.

Similarly Participant 25 explained, ‘it’s good. I mean it’s a therapeutic exercise for me as well you know.’

It was clear that participants were grateful to be given an opportunity to discuss their experiences and the impact that being FTP a woman had had upon their lives, as well as being appreciative that this form of sexual violence was being recognised and studied. Some of the men had never discussed their experience(s) prior to their interview, and this study provided them with a ‘safe’ opportunity to tell their story. Furthermore, ‘participating in the research validated [participants’] interpretation of [their experiences]’ and they ‘understood [they were] not alone in [their] suffering’ (Bergen, 1993, p. 209) or their experiences. This was a sentiment echoed by Participant 21, who explained, ‘that helps, that people know it’s not just them.’ The men also saw the research as being of political, practical and legal importance, and were hopeful that sharing their experiences would have a positive impact on societal, legal and criminal justice responses for other survivors. For example, Participant 1 said:

I’m glad that I can participate in this … if it can help to change somebody’s mind about what happens, even if I can’t put my perpetrator behind bars that would at least be something. If somebody else’s perpetrator can be put behind bars then … I’ve actually changed something.

This is something that has been seen in research on VAWG, where whilst for female participants it may be too late to alleviate their suffering directly, their participation ‘can contribute to legislation, policy, or the behaviour of agencies in ways which later enhance the experience of others’ (Maynard, 1994, p. 17).

Several participants contacted us following their interview to express the importance of their involvement and to thank us for conducting the research. For example, Participant 29's email read: ‘Thanks for doing this work and raising awareness about this issue. I’d forgotten how much what happened to me had affected my life and it’s a serious issue that needs more exposure.’ Participant 7 also contacted us to express the effects that participation had upon him:

Speaking to your researcher was a powerfully positive experience for me – speaking to someone other than a counsellor about my experiences did feel like a weight being lifted, perhaps speaking in a non-clinical environment helped me to come to terms with it all as not being something wrong with me. I would like to thank you for this research project for shining a light on the issue and, personally, for the difference it has made to me.

The extent and depth of the gratitude expressed by participants was something that we were not expecting, and reinforced to us the importance of conducting this research, as well the impact for participants of being involved. For other researchers who are conducting, or are considering conducting, similar research studies with male survivors, this reinforces the importance of ‘giving them a voice’, of involving them as co-producers of knowledge, and of being aware of the ethical and methodological considerations that may emerge. This will help to ensure that participants’ experiences are positive and beneficial.

## Key Recommendations

Reflecting the discussions in this article and our experiences interviewing male survivors of sexual violence, there are a number of important recommendations that should be considered by those conducting similar research in the future.

First, it is important to recognise that male survivors represent a hidden group of sexual violence victims, and therefore engaging their participation in research studies could be challenging. To encourage as many men as possible to share their experiences, researchers should use a variety of approaches to engage male participants. These include the use of mainstream and social media, working with specialist male survivor support services, and creating a project website that potential participants can access. Provide as much detail upfront as possible to potential participants, including contact details for researchers so they can get in touch with questions or concerns about their involvement, and signpost to specialist male survivor support services throughout.

Secondly, it is important to be flexible in the interview process and recognise that participants may not have disclosed their experiences before. Whilst we took a semi-structured approach to the interviews, we found that the men we interviewed appreciated being allowed to share their stories uninterrupted at the beginning of the interview. We then followed up with more specific questions when appropriate. It is important to ensure that participants are signposted to specialist male support services both prior to, and following, their interview. Ensure that processes are put in place and communicated with participants in relation to their safety, for example what would happen if calls were disconnected. Based on our experiences, we advise that it would be useful to put a ‘triage’ system in place for those participants who may be particularly distressed as a result of their interview.

Thirdly, be aware of the impact that the researcher’s gender may have on the interview process. We recommend that research projects which involve interviewing male survivors should include at least one female researcher, as participants in our study generally indicated a preference to talk to women about their experiences. Where research teams are mixed-gendered, participants should be given the option to choose the gender of the interviewer prior to their interview. All researchers should be aware of and reflect upon the fluidity of power dynamics within interviews. Female researchers in particular should be aware of the challenges they may face interviewing men (as noted within existing scholarship). However, they should not assume that any or all of these will emerge within interviews they conduct.

Fourthly, researchers must remember the importance of the research to participants. The voices of male survivors should be centralised throughout empirical research projects and they should be involved as co-producers of knowledge. Being aware of ethical and methodological considerations that may emerge will help to ensure that participants experiences are as positive as possible.

Finally, in our experience, partnering with Survivors Manchester was central to the success of the project for all involved. This was particularly the case for the male survivor participants who benefitted both directly and indirectly from Survivors Manchester’s involvement in the ways outlined at several points in this article. VAWG researchers have recommended partnerships with support services for women (e.g Ellsberg & Heise, 2005, p. 45), and similarly we recommend that all research projects which involve interviewing male survivors of sexual violence partner with a *specialist male survivor support organisation*. By this, we mean an organisation that either: (a) offers specialist male-specific counselling and support solely to male survivors; or (b) offers counselling and support to both male and female survivors, but offers specialist male-specific counselling and support to male survivors, and offers them access to the service on an equal basis to female survivors. Examples of such organisations include those that have been accredited as meeting the *Quality Standards for Services Supporting Male Survivors of Sexual Violence* (Male Survivors Partnership, n.d.). We are very specific on this requirement because many support services exclude male survivors, and others, whilst claiming to offer support, provide them with a more limited service than that provided to female survivors. By partnering with a specialist male survivor support service, and collaborating with them at every stage of the project, the risks of harm for survivor participants can be minimised, and the benefits and impacts of the research for male survivors more widely can be maximised.

## Concluding Thoughts

In this article we have not covered every methodological and ethical aspect of a research project involving interviews with male survivors of female-perpetrated sexual violence, but have drawn out what we believe to be four of the key issues and considerations that emerged for us. In considering these issues we have drawn upon literature from cognate and related areas of study, particularly on interviewing female survivors of VAWG crimes and interviewing men more broadly, which has allowed us to critically interrogate these issues and provide reflections for researchers considering empirical studies with male survivors. Whilst engaging with such literature is undoubtedly useful in the context of interviewing male survivors of sexual violence, the absence of literature on interviewing this specific group of men is problematic given the prevalence of male sexual violence victimisation, and the growing body of scholarship on this issue. Whilst this article has gone some way to filling a gap in the literature and supporting the development of knowledge and understanding, it is clear that further exploration of ethical and methodological considerations associated with engaging male survivors as co-producers and participants in research are needed. In closing this article, we would like to call for other researchers who are conducting empirical research with male survivors to share their experiences, so as to allow for continued reflection upon, and development of, methodological and ethical practices when working with this group of men.
